# Efficacies of Controlling Morning Blood Pressure and Protecting the Kidneys by Treatment With Valsartan and Nifedipine CR or Valsartan and Amlodipine (MONICA Study)

**DOI:** 10.4021/jocmr1563w

**Published:** 2013-10-12

**Authors:** Tohru Tanaka, Shin-ichiro Miura, Masatoshi Tanaka, Yoshinari Uehara, Tadashi Hirano, Keijiro Saku

**Affiliations:** a Department of Cardiology, Fukuoka University School of Medicine, Fukuoka, Japan; b Tanaka Clinic, Kitakyusyu, Japan; c Mihagino Hospital, Kitakyusyu, Japan; d Department of Molecular Cardiovascular Therapeutics, Fukuoka University School of Medicine, Fukuoka, Japan

**Keywords:** Combination therapy, Morning hypertension, Albuminuria, Nifedipine CR, Amlodipine

## Abstract

**Background:**

It is controversial whether a single-pill fixed-dose combination of angiotensin II type 1 receptor blocker and calcium channel blocker (CCB) is effective for all types of hypertension.

**Methods:**

Thirty-five patients with uncontrolled blood pressure (BP) under treatment with valsartan 80 mg/day or amlodipine 5 mg/day were enrolled. They were randomly divided into two treatment groups: a single-pill fixed-dose combination of valsartan 80 mg/day and amlodipine 5 mg/day in the morning (VA group), or valsartan 80 mg/day in the morning and nifedipine CR 20 mg/day at night (VN group), and treated for 16 weeks. If the patient did not reach the target office BP at 8 weeks, they received double doses of CCBs.

**Results:**

In the VN group, morning diastolic BP was significantly lower than the respective values in the VA group at 8 weeks. The percentage of patients who required a double dose of CCB in the VN group was significantly lower than that in the VA group. At 16 weeks, the BP levels in both groups were significantly reduced. Urinary albumin/creatinine at 16 weeks was significantly less than that at 0 weeks in the VN group.

**Conclusion:**

Combination therapy with valsartan and nifedipine CR may help to control morning BP and protect the kidneys.

## Introduction

Although better blood pressure (BP) control is associated with remarkable clinical benefits with regard to cardiovascular (CV) and renal protection, many patients still show higher BP after treatment with medium-dose angiotensin II type 1 receptor blockers (ARBs) or calcium channel blockers (CCBs). Most patients with hypertension (HT) require two or more drugs to achieve their target BP [1], and various guidelines recommend a combination of ARBs and CCBs [2, 3]. Recently, many single-pill fixed-dose combinations of ARBs and CCBs have become available for clinical use in Japan and have been shown to be helpful for controlling BP [4].

Morning HT can lead to progressive target organ damage and trigger CV events [5, 6]. Antihypertensive treatment with a target morning BP of < 135/85 mmHg leads to strict 24-hour BP control, which should achieve more effective protection than conventional antihypertensive treatment based on the office BP [5]. Although treatments for morning HT include the administration of long-acting CCBs, such as amlodipine, in the morning, this does not necessarily confer the target BP. Some ingenuity is required to achieve the target BP, such as the combination of CCBs and ARBs or the administration of antihypertensive agents at bedtime.

Patients with chronic kidney disease (CKD) are at significantly higher risk of CV disease (CVD) [7, 8], and patients with overt proteinuria as well as albuminuria without a reduction in the estimated glomerular filtration rate (eGFR) are also at significantly higher risk [8]. Proteinuria or albuminuria itself should be a target for reducing hard end points. CCBs are some of the most often prescribed medications for the prevention of albuminuria, next to renin-angiotensin system blockers. Nifedipine has been shown to prevent increases in albuminuria in normotensive patients and to decrease albuminuria in hypertensive patients [9-12]. The effects of nifedipine were comparable to those of the angiotensin converting enzyme inhibitor perindopril. On the other hand, amlodipine did not decrease proteinuria in patients with HT [13].

Six single-pill fixed-dose combinations of ARBs/CCBs are available for clinical use in Japan. It is controversial whether treatment with a single-pill fixed-dose combination is effective for all types of HT, such as morning HT, or in patients with albuminuria. A single-pill fixed-dose combination of valsartan (80 mg/day) and amlodipine (5 mg/day) is the best-selling and a standard single pill in Japan. As mentioned before, nifedipine CR is also a long-acting CCB in Japan and decreased urinary albumin (U-Alb) levels [9-12], whereas amlodipine did not decrease proteinuria [13]. In addition, morning BP levels were significantly higher in patients with albuminuria than in patients without albuminuria [14]. We hypothesized that the administration of valsartan in the morning and nifedipine CR at night may be useful for controlling morning HT and decreasing albuminuria compared to a standard single-pill (valsartan (80 mg/day) and amlodipine (5 mg/day)) in the morning. Therefore, in this study, we compared the efficacies of two kinds of treatment (a single-pill fixed-dose combination of valsartan and amlodipine in the morning, or valsartan in the morning and nifedipine CR at night) at controlling morning BP and protecting the kidneys.

## Methods

### Study design

Thirty-five hypertensive patients (18 male and 17 female, 72 ± 13 years) who had uncontrolled BP according to the Japanese Society of Hypertension Guidelines for the Management of Hypertension 2009 (JSH2009) [1] despite treatment with a medium dose of valsartan (80 mg/day) or amlodipine (5 mg/day) were enrolled. They were randomly divided into two treatment groups (a single-pill fixed-dose combination of valsartan (80 mg/day) and amlodipine (5 mg/day) in the morning (n = 19, VA group), or valsartan (80 mg/day) in the morning and nifedipine CR (20 mg/day) at night (n = 16, VN group)) after adjusting several factors (age, gender, systolic BP (SBP) and U-Alb/U-creatinine (Cr)). Pretreatment with valsartan or amlodipine changed either treatment. Office and morning SBP and diastolic BP (DBP) and pulse rate (PR) measurements were obtained at 0, 4, 8, 12 and 16 weeks. Morning BP was measured at least 2 times within 1 h after waking up, after urination, before dosing in the morning, before breakfast according to the JSH2009. On the other hand, office BP is measured after dosing around 10 am. If the patients did not reach the target office BP at 8 weeks, they were to receive double doses of CCBs (patients in the VA and VN groups received amlodipine (10 mg/day) in the morning and nifedipine CR (40 mg/day) at night, respectively). We excluded patients with secondary HT, heart failure, liver dysfunction, renal dysfunction (defined as a serum Cr level of more than 2.0 mg/dL), pregnancy, or a history of allergy to ARBs and/or CCBs. The protocol in this study was approved by the ethics committee of Fukuoka University Hospital, and all subjects gave their informed consent to participate.

### Evaluation of clinical parameters

BP was determined as the mean of two measurements obtained in an office setting by the conventional cuff method using a mercury sphygmomanometer after at least 5 minutes of rest. Body mass index (BMI) was calculated as weight (kg)/height (m)^2^.

We analyzed blood and urinary levels of biochemical parameters at 0 and 16 weeks. All of the blood and urinary samples were collected in the morning after the patients had fasted overnight. Data regarding serum levels of biochemical parameters, such as high-density lipoprotein-cholesterol (HDL-C), low-density lipoprotein-cholesterol (LDL-C), triglycerides (TG), Cr, fasting blood glucose (FBS) and hemoglobin A1c (HbA1c), uric acid (UA), sodium (Na), potassium (K), eGFR, high-sensitive C reactive protein (hs-CRP) and cystatin C (Cys-C), were collected in all patients. Plasma samples were immediately stored at -80 °C for the subsequent assay of pentraxin-3 (PTX-3) and monocyte chemotactic protein-1 (MCP-1) levels by enzyme-linked immunosorbent assay. The concentration of plasma PTX-3 or MCP-1 showed a coefficient of variation of < 5%.

The characteristics of the patients, with regard to history of DL, diabetes mellitus (DM), HU, smoking status and medication use, were obtained from medical records. Patients with LDL-C ≥ 140 mg/dL, TG ≥ 150 mg/dL, and/or HDL-C < 40 mg/dL, or who were receiving lipid-lowering therapy, were considered to have DL. DM was defined using the American Diabetes Association criteria or the use of a glucose-lowering drug. Hyperuricemia (HU) was defined as a serum UA level of ≥ 7.0 mg/dL or the use of uric acid-lowering drugs.

### Statistical analysis

Statistical analysis was performed using the Stat View statistical software package (Stat View 5; SAS Institute Inc., Cary, NC, USA). We performed intension-to-treat analysis. Categorical variables were compared between groups by a chi-square analysis. Significant changes in continuous variables during the study period were examined by Student’s unpaired t-test or Wilcoxon’s rank-sum test. Data are shown as the mean ± standard deviation (S.D.). A P value of less than 0.05 was considered to reflect significance.

## Results

### Patient characteristics

Thirty-five patients were enrolled and randomly divided into the VA (n = 19) and VN (n = 16) groups. Table 1 showed the clinical characteristics of the total 35 patients, who consisted of 18 (51 %) males. Five patients withdrew during the study period because of hypotension (2 in the VN groups), implantation of a pacemaker (1 in the VN group) and not visiting the hospital (1 each in the VA and VN groups, respectively). Patients had taken valsartan (n = 29, dose 80 mg/day) or amlodipine (n = 6, 5 mg/day) before the study. The incidences of several coronary risk factors such as gender, BMI, smoking and DL, but not DM, were similar in the VA and VN groups. There was no significant difference in the use of medications such as β-blockers and statin, except for sulfonyl urea (SU), between the groups. We did not change these medications throughout the study.

**Table 1:** Baseline Patient Characteristics

	VA group (n = 19)	VN group (n = 16)
Age, year	71 ± 14	74 ± 11
Male, %	47	56
BMI, kg/m^2^	23 ± 5	23 ± 3
Smoking, %	21	6
DM, %	11	44*
DL, %	53	63
HU, %	21	19
Office measurement		
SBP, mmHg	158 ± 11	158 ± 11
DBP, mmHg	86 ± 13	81 ± 10
PR, /min	70 ± 12	68 ± 11
Morning measurement		
SBP, mmHg	157 ± 12	152 ± 10
DBP, mmHg	90 ± 16	80 ± 13
PR, /min	67 ± 9	72 ± 10
Medication		
β-blocker, %	11	19
Statin, %	17	21
α-Gl, %	0	13
SU, %	0	25*
DPP-4 inhibitor, %	0	13

BMI, body mass index; DM, diabetes mellitus; DL, dyslipidemia; HU, hyperuricemia; SBP, systolic blood pressure; DBP, diastolic blood pressure; PR, pulse rate; α-GI, α-glycosidase inhibitor; BG, biguanide; SU, sulfonyl urea; DPP-4, dipeptidyl peptidase-4.

### Time course of office and morning BP levels

**Figure 1:**
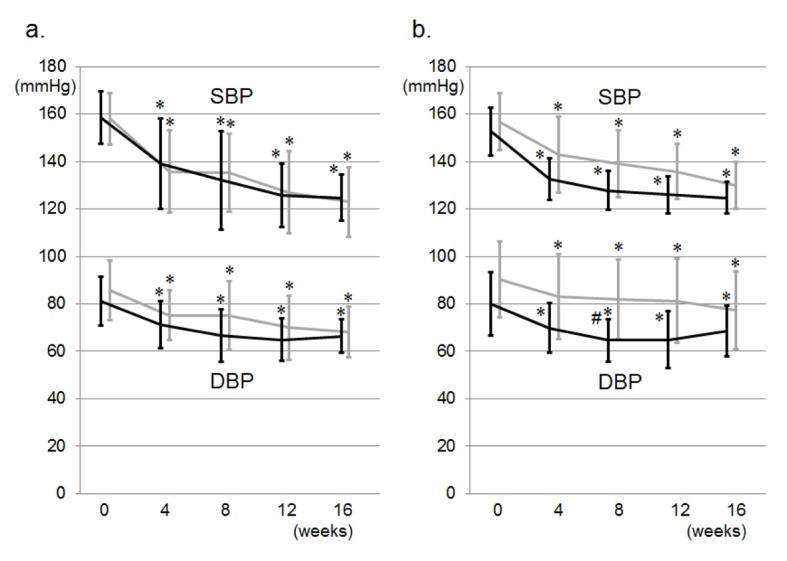
Changes in office SBP and DBP (a) and morning SBP and DBP (b) during the study period in the VA (gray lines) and VN (black lines) groups. *P < 0.05 vs. at 0 weeks. #P < 0.05 vs. VA group.

**Figure 2:**
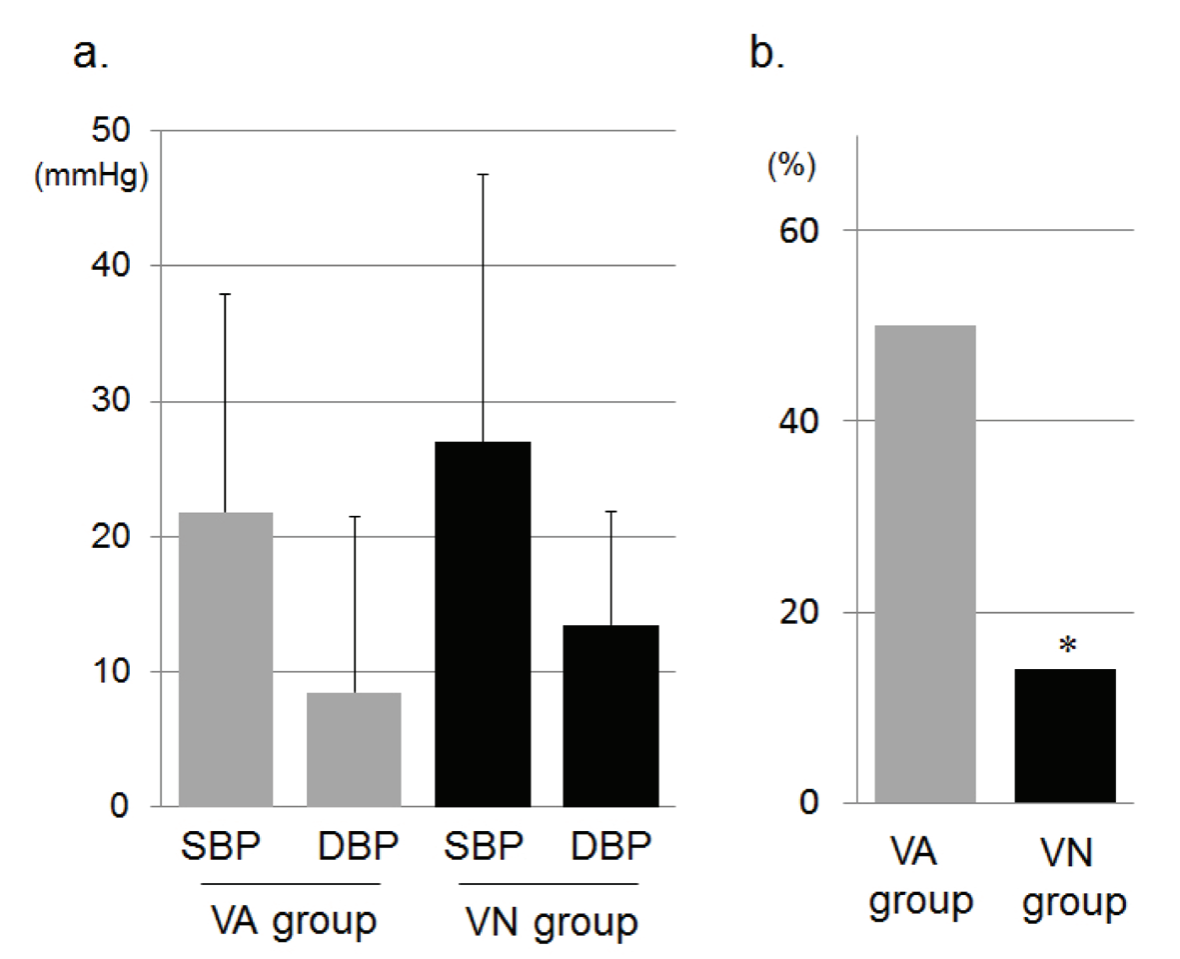
a). Reductions of office SBP and DBP from 0 weeks to 8 weeks in the VA and VN groups. b). Percentages of patients who required an increase in the dose of CCB at 8 weeks in the VA and VN groups. *P < 0.05 vs. VA group.

Figure 1a shows changes in office BP during the study period. There was no difference in office BP at 0 weeks between the VA and VN groups. SBP and DBP were significantly decreased in both groups at 8 weeks. The reductions in SBP and DBP in the VN group were greater than those in the VA group, but these differences were not significant (Fig. 2a). Two and 9 patients in the VN and VA groups, respectively, did not reach the target office BP and therefore the doses of CCBs were increased: the percentage of patients in the VN group (14%) was significantly lower than that in the VA group (50%) (Fig. 2b). Although there were significant differences in the percentage of DM and SU use, these factors did not associate with the percentage of patients who were to receive double doses of CCBs (data not shown). At 16 weeks, there were no differences in the reduction of office BP between the groups, and the BP levels in both groups were significantly reduced. Figure 1b shows changes in morning BP during the study period. In the VN group, morning SBP tended to be lower and morning DBP was significantly lower than the respective values in the VA group at 8 weeks, although there were differences in SBP (5 mmHg) and DBP (10 mmHg) between the VN and NA groups at 0 weeks, but not significantly. At 16 weeks, there were no differences in office or morning BP between the groups, and the office and morning BP levels in both groups were significantly reduced (Fig. 1a, b). In addition, there were no changes in office or morning PR between the groups or between 0 and 16 weeks throughout the study period.

### Changes in biochemical parameters

Biochemical parameters in blood at 0 and 16 weeks are shown in Table 2. There were no differences in the levels of biochemical parameters at 0 weeks between the VA and VN groups. All of the parameters, such as liver function, lipid profile and electrolyte at baseline and after 16 weeks, were similar between the VA and VN groups. In addition, there were no differences in the levels of markers of inflammation, such as hs-CRP, MCP-1 and PTX-3, between the VA and VN groups at 0 weeks, and there were no significant changes after 16 weeks.

**Table 2:** Biochemical Parameters in Blood at 0 and 16 Weeks

	VA		VN	
	0 weeks (n = 19)	16 weeks (n = 18)	0 weeks (n = 16)	16 weeks (n = 12)
AST, IU/L	26 ± 13	25 ± 6	26 ± 13	26 ± 10
ALT, IU/L	19 ± 7	19 ± 7	24 ± 15	19 ± 9
BUN, mg/dL	16 ± 5	18 ± 5	16 ± 4	16 ± 4
Cr, mg/dL	0.9 ± 0.3	1.0 ± 0.3	0.9 ± 0.1	0.9 ± 0.2
eGFR, mL/min/1.73m^2^	58 ± 18	50 ± 16	61 ± 11	59 ± 8
UA, mg/dL	5.5 ± 1.2	5.8 ± 1.0	5.4 ± 1.2	5.0 ± 1.1
Cl, mEq/L	105 ± 2	103 ± 4	103 ± 4	102 ± 2
Na, mEq/L	142 ± 3	141 ± 3	141 ± 4	141 ± 4
K, mEq/L	4.4 ± 0.6	4.1 ± 0.8	4.2 ± 0.5	4.2 ± 0.5
LDL-C, mg/dL	104 ± 39	100 ± 39	117 ± 21	119 ± 27
HDL-C, mg/dL	62 ± 18	58 ± 20	57 ± 20	58 ± 16
TG, mg/dL	157 ± 146	173 ± 157	166 ± 114	154 ± 75
HbA1c, %	5.6 ± 0.4	5.5 ± 0.6	5.9 ± 0.8	6.5 ± 1.5
hs-CRP, mg/dL	0.15 ± 0.19	0.12 ± 0.12	0.07 ± 0.06	0.08 ± 0.06
MCP-1, pg/mL	456 ± 170	432 ± 133	394 ± 135	447 ± 285
PTX-3, ng/mL	2.3 ± 1.0	2.0 ± 1.2	2.2 ± 0.9	2.2 ± 1.0

AST, aspartate aminotransferase; ALT, alanine aminotransferase; BUN, blood urea nitrogen; Cr, creatinine; eGFR, estimated glomerular filtration rate; UA, uric acid; Cl, chloride; Na, sodium; K, potassium; LDL-C; low-density lipoprotein cholesterol; HDL-C, high- density lipoprotein cholesterol; TG, triglyceride; hs-CRP, high-sensitive C-reactive protein; MCP-1,monocyte chemotactic protein-1; PTX-3, pentraxin-3.

**Figure 3:**
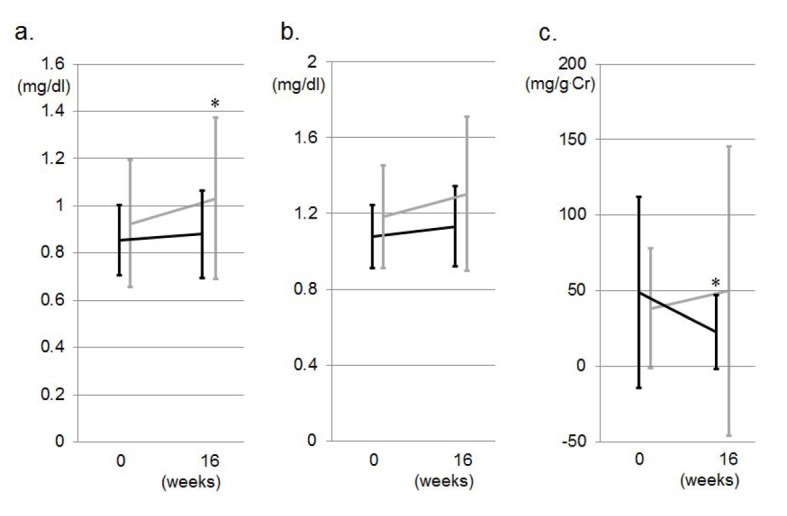
Markers of renal function, such as serum Cr (a), Cys-C (b) and U-Alb/U-Cr (c), during the study period in the VA (gray lines) and VN (black lines) groups. *P < 0.05 vs. at 0 weeks.

Markers of renal function, such as serum Cr, Cys-C and U-Alb/U-Cr, are shown in Figure 3. The U-Alb/U-Cr at 16 weeks (23 ± 24 mg/g.Cr) was significantly less than that at 0 weeks (49 ± 63) in the VN group, but not in the VA group (39 ± 39 at 0 weeks and 50 ± 95 at 16 weeks). Moreover, serum Cr at 16 weeks (1.03 ± 0.34 mg/dL) was significantly greater than that at 0 weeks (0.92 ± 0.27) in the VA group, but not the VN group, whereas there were no differences in the levels of Cys-C between 0 and 16 weeks or between the two groups.

### Associations between changes in SBP or DBP and U-Alb/U-Cr

Since U-Alb/U-Cr significantly decreased in the VN group, we analyzed the associations between changes in office BP and changes in U-Alb/U-Cr (ΔU-Alb/U-Cr = the values at 16 weeks minus the values at 0 weeks). ΔU-Alb/U-Cr was not significantly correlated with Δoffice SBP (r = 0.120, P = 0.548) or Δoffice DBP (r = 0.119, P = 0.588). In addition, ΔU-Alb/U-Cr was not associated with Δmorning SBP (r = -0.349, P = 0.185) or Δmorning DBP (r = -0.313, P = 0.237). In addition, although there were significant differences in the percentage of DM and SU use, these factors did not associate with ΔU-Alb/U-Cr (data not shown).

### Safety and tolerability

As described previously, some patients experienced adverse events, such as hypotension (2 patients in the VN group and none in the VA group) and pacemaker implantation (1 in the VN group), during the study period. Adverse events that were considered to be treatment-related were infrequent in both groups. In addition, there were no remarkable findings of clinical concern regarding the biochemical parameters or vital signs.

## Discussion

The present results suggest that combination therapy consisting of valsartan in the morning and nifedipine CR at night may be more useful for controlling morning BP and protecting the kidneys than combination therapy with amlodipine and valsartan in the morning. Both combinations were associated with a significant reduction in BP at 16 weeks when patients received double doses of CCBs if they did not reach the target office BP at 8 weeks. Combination therapy with valsartan and nifedipine CR, but not that with valsartan and amlodipine, significantly prevented albuminuria after treatment independent of the reduction of BP.

Although both the VA and VN groups showed a significant reduction in BP at 16 weeks, a significantly greater percentage of patients in the VA group required an increase in the dose of CCB. There are several possible explanations for this difference. First, although amlodipine has a relatively long elimination half-life of 35 to 45 hours [15], amlodipine was administered in the morning in the VA group. On the other hand, nifedipine CR was administered at night. The administration of CCBs at different times also influences BP control, and particularly morning BP. In fact, the reduction in BP after treatment with nifedipine at bedtime was significantly greater mainly during sleep at night [16]. The morning increase in BP was significantly reduced only after the administration of nifedipine at bedtime. Meng et al also reported that, compared to the concomitant administration of amlodipine and fosinopril in the morning, administration of the drugs at different times significantly decreased nocturnal BP and normalized the circadian BP pattern [17]. In fact, Hermida et al indicated that valsartan/amlodipine combination therapy should be preferably administered at bedtime [18]. Second, not all CCBs have the same effects, and some benefits conferred by CCBs may not be class effects. Significant reductions in BP were noted after amlodipine was switched to nifedipine CR in elderly patients with HT [19]. A significantly higher percentage of patients in the nifedipine CR treatment group achieved their target BP, compared to the amlodipine treatment group [20]. Nifedipine CR had a stronger antihypertensive effect than amlodipine on morning and office BP [21]. On the other hand, the depressor effect of nifedipine CR was comparable to that of amlodipine [22]. The depressor effect of nifedipine CR may be similar to or superior to that of amlodipine, although different doses of nifedipine and amlodipine were used in each study. The differences in the depressor effect may affect the percentage of patients who required an increase in the dose of CCB in the VA group. In a recent experiment, the effect of the inhibition of aldosterone-induced activation of mineralocorticoid receptor by nifedipine was superior to that by amlodipine, indicating that nifedipine might provide better BP control [23]. In addition, although we did not analyze the outcome of CV events, fewer CV events were seen when patients took ≥ 1 antihypertensive medications at bedtime compared to when they took all of their medications in the morning, among patients with CKD [24].

Combination therapy with valsartan and nifedipine CR, but not valsartan and amlodipine, significantly decreased albuminuria. There are three possible explanations for why combination therapy with nifedipine CR and valsartan was useful. First, the administration of CCBs at different times may influence albuminuria. Amlodipine was administered in the morning in the VA group, whereas nifedipine CR was administered at night. When nifedipine CR was administered at night, nocturnal BP was significantly decreased [17], and U-Alb excretion may also be decreased. Second, different kinds of CCBs prevent albuminuria to different degrees [11, 12]. In fact, some reports have indicated that nifedipine CR decreased albuminuria. Nifedipine CR and cilnidipine, but not efonidipine and amlodipine, significantly reduced albuminuria [11]. Combination therapy with standard-dose candesartan and nifedipine CR is more effective than up-titrated candesartan monotherapy for reducing BP and improving U-Alb while maintaining eGFR [12]. Interestingly, nifedipine prevented pressure-induced afferent arteriolar vasoconstriction in an isolated perfused hydronephrotic kidney in rat [25]. Moreover, nifedipine also dilated afferent, as well as efferent arterioles [26]. Third, nifedipine has been shown to preserve endothelial function in patients with hypertension and/or coronary artery disease [27, 28]. Since microalbuminuria in diabetic patients, as well as nondiabetic individuals, is associated with endothelial dysfunction [29, 30], nifedipine might decrease U-Alb through the improvement of endothelial function in the kidney.

In this study, there were no significant changes in the levels of inflammation markers after treatment, although both combinations were associated with a significant reduction of BP. ARBs have been shown to decrease inflammation markers, such as CRP and PTX-3 [31, 32], and most of the patients received ARBs before entering this study. In addition, microalbuminuria is also associated with low-grade inflammation [33]. Although nifedipine decreased U-Alb/U-Cr in this study, it did not decrease inflammation markers. Further studies will be needed to resolve this issue.

### Study limitations

This study has several important limitations. First, the sample size was relatively small, which limits our ability to determine significance. Second, we applied a changeover design with switching from valsartan or amlodipine to 2 combination therapies. However, we randomly divided the patients into two groups and there was no significant difference in baseline BP between the groups. In addition, we should also better to compare the efficacies of the single-pill fixed-dose combination of varsartan and amlodipine in the morning with those of the single-pill in the evening. Third, although 24 hours ambulatory monitoring is useful for measuring morning BP, we did not use it. Finally, 24 hour urinary excretion is much better method to evaluate albuminuria.

### Conclusions

Combination therapy consisting of valsartan in the morning and nifedipine CR at night may be more useful for controlling morning BP and protecting the kidneys than the combination of valsartan and amlodipine in the morning.
